# Genome annotation of *Anopheles gambiae *using mass spectrometry-derived data

**DOI:** 10.1186/1471-2164-6-128

**Published:** 2005-09-19

**Authors:** Dário E Kalume, Suraj Peri, Raghunath Reddy, Jun Zhong, Mobolaji Okulate, Nirbhay Kumar, Akhilesh Pandey

**Affiliations:** 1 McKusick-Nathans Institute of Genetic Medicine and Department of Biological Chemistry and Oncology, Johns Hopkins School of Medicine, Baltimore, MD 21205, USA; 2 Department of Biochemistry and Molecular Biology, University of Southern Denmark, Odense, DK-5230, Denmark; 3 Department of Molecular Microbiology and Immunology, Johns Hopkins Malaria Research Institute, Johns Hopkins Bloomberg School of Public Health, Baltimore, MD 21205, USA; 4 Institute of Bioinformatics, Discoverer Unit 1, 7th Floor International Tech Park Ltd., Whitefield Road, Bangalore – 560 066, India

## Abstract

**Background:**

A large number of animal and plant genomes have been completely sequenced over the last decade and are now publicly available. Although genomes can be rapidly sequenced, identifying protein-coding genes still remains a problematic task. Availability of protein sequence data allows direct confirmation of protein-coding genes. Mass spectrometry has recently emerged as a powerful tool for proteomic studies. Protein identification using mass spectrometry is usually carried out by searching against databases of known proteins or transcripts. This approach generally does not allow identification of proteins that have not yet been predicted or whose transcripts have not been identified.

**Results:**

We searched 3,967 mass spectra from 16 LC-MS/MS runs of *Anopheles gambiae *salivary gland homogenates against the *Anopheles gambiae *genome database. This allowed us to validate 23 known transcripts and 50 novel transcripts. In addition, a novel gene was identified on the basis of peptides that matched a genomic region where no gene was known and no transcript had been predicted. The amino termini of proteins encoded by two predicted transcripts were confirmed based on N-terminally acetylated peptides sequenced by tandem mass spectrometry. Finally, six sequence polymorphisms could be annotated based on experimentally obtained peptide sequences.

**Conclusion:**

The peptide sequences from this study were mapped onto the genomic sequence using the distributed annotation system available at Ensembl and can be visualized in the context of all other existing annotations. The strategy described in this paper can be used to correct and confirm genome annotations and permit discovery of novel proteins in a high-throughput manner by mass spectrometry.

## Background

The recent completion of *Anopheles gambiae *genome sequence [1] provided an architectural scaffold for mapping, identifying, selecting, and exploiting malaria insect vector genes for future studies. *An. gambiae *genome consists of 3 pairs of chromosome, designated as 2R/2L, 3R/3L and X. The Y chromosome is yet to be completely sequenced and assembled because of the high number of transposable element fragments. Thus far, approximately 85% of the genome has been assembled with the total genome size being 278 Mbp. About 15,189 genes are annotated in the *An. gambiae *genome, of which 11,757 are derived from prediction programs [2]. Currently, there are approximately 700 known *An. gambiae *proteins that are annotated in the databases. The annotation of the *An. gambiae *genome sequence has been an ongoing process since it was completed in 2002 [1]. The assembled genome is publicly available through NCBI (National Center for Biotechnology Information) and EBI (European Bioinformatics Institute)/Ensembl . It is important to note that the existing genome annotation is mainly based on *de novo *gene predictions in addition to a small number of experimentally obtained transcripts. Because of the magnitude of sequence data, automatic annotation is a necessity. However, this results in different types of errors, some of which can be overcome by combining manual annotation and experimental evidence. In this regard, mass spectrometry is a powerful tool that can contribute to the identification of novel genes and assist in confirmation of predicted transcripts as well as correction of incorrect assignments from automated gene annotations [3]. The use of mass spectrometry to assist the validation of genome annotation has been previously demonstrated in prokaryotes [4], yeast [5], plants [6] and humans [7]. However, two of these studies [5,7] did not directly search mass spectrometry-derived data against the genomic databases – rather, a post hoc integration of peptide sequences with the genomic sequence was carried out. This approach is not preferable for annotating genomes because if there is any region of a genome that has no transcript associated with it (e.g. introns and intergenic regions), it will not be identified.

In this study, we carried out a proteomic analysis of salivary gland proteins from *An. gambiae *and searched this data against the *An. gambiae *genome database. We were able to validate 73 transcripts, which were predicted from the genome. We also corrected several erroneous predictions (e.g. missed exons) and identified one gene that was not predicted at all. To share our results with the biomedical community, we have taken advantage of the Distributed Annotation System [8] provided by Ensembl. We have mapped all the peptides identified in this study such that they can be visualized by anyone using the Ensembl genome browser. It is hoped that availability of additional proteomic data will aid in further refining the annotation of genomic data.

## Results and discussion

### Curent status of the *An. gambiae *genome sequence and annotation

**Figure 1:**
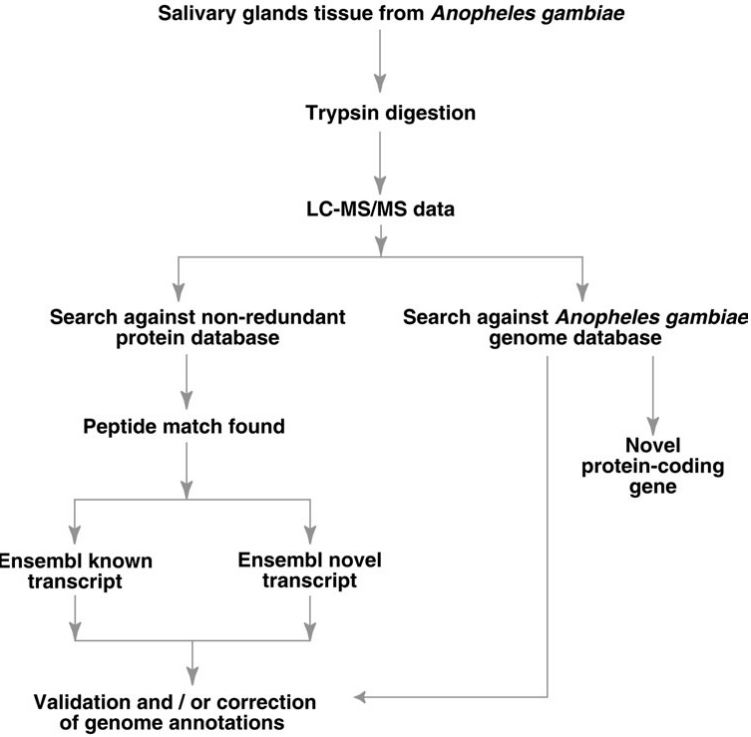
**A workflow depicting the steps involved in mass spectrometry data analysis for genome annotation purposes. **In this case, the tryptic peptide mixture derived from digestion of *Anopheles gambiae *salivary gland proteins was analyzed by liquid chromatography tandem mass spectrometry (LC-MS/MS). The mass spectrometry data was searched against the NCBI non-redundant protein database to identify known or novel transcripts from *An. gambiae*. The data was also searched against the *An. gambiae *genome database to identify novel protein-coding genes. A careful bioinformatics analysis was performed to use peptide data for correcting genomic annotations.

A preliminary annotation of protein coding regions of the *An. gambiae *genome has recently been published. Using automated gene prediction programs, two groups have annotated a total of 15,189 predicted transcripts [1]. This included a total of 7,840 predicted transcripts that were unique to Otto, the gene prediction pipeline used by the Celera group and 1,375 predicted transcripts that were unique to the Ensembl annotation [1]. Altogether, 5,974 transcripts were predicted by both analyses. It must be cautioned, however, that these are preliminary annotations for the *An. gambiae *genome. A validation of predicted transcripts could be accomplished through the use of direct peptide sequence data such as that obtained by tandem mass spectrometry in our study. In this study, we have used the strategy outlined in Figure 1 to map the peptides identified by mass spectrometry onto the existing Ensembl annotations of the *Anopheles *genome. We will illustrate seven different situations in which mass spectrometry data assisted us in the genome annotation: a) peptide sequences that matched exons in known transcripts; b) peptide sequences that matched exons in novel transcripts; c) peptide sequences that matched regions of the genome where no genes were predicted at all; d) matching of peptide sequences regions annotated as untranslated regions (UTRs); e) matching of peptide sequences to regions annotated as introns of known or novel genes; f) data on N-terminal acetylation sites for mapping the amino terminus of the mature protein; and, g) sequence polymorphisms that could represent coding single nucleotide polymorphisms (cSNPs).

### Use of distributed annotation system at Ensembl for sharing our genome annotation with the community

**Figure 2:**
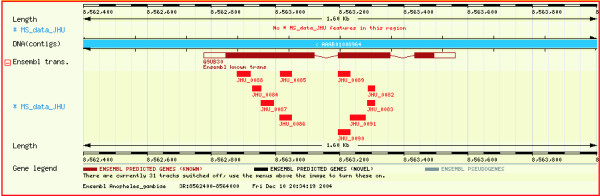
**A screenshot depicting the mapping of mass spectrometry-derived peptide sequence data onto the genome sequence in 'ContigView' of Ensembl Genome Browser**. The red rectangles on 'MS data JHU' track are peptide sequences obtained through tandem mass spectrometry. The brown colored rectangles on the 'Ensembl trans.' are the Ensembl known transcripts. Ten peptides are shown to match two exons in the known transcript encoding D7-related protein 1. The prefix JHU refers to Johns Hopkins University and is followed by a unique identifier for each peptide. Clicking on the peptide accession number links to a page  containing additional information including its sequence.

DAS [8] allows integration of annotation efforts from various sources and facilitates visualization of annotations interactively in a browser. It serves as a means for decentralization of annotation efforts. A DAS reference server providing access to the *An. gambiae *genome is available through Ensembl. Therefore, we decided to take advantage of sharing our annotations of the *An. gambiae *genome with the community using this resource. We uploaded all the peptides identified in our effort onto the Ensembl DAS server. They can now be viewed as peptide sequence hits that mapped to specific genomic sequences along with all other annotations provided by Ensembl (ESTs, transcript, proteins etc.). To view the peptide data, a user has to select the 'MS data JHU' option under 'DAS Sources' pull down menu in the 'Detailed View' panel in ContigView page of Ensembl. Figure 2 shows a screenshot of the DAS view of ten peptides that were sequenced by our laboratory under the 'MS data JHU' track in the negative strand. As indicated in the screenshot, there were no peptide matches in the positive strand. We mapped a total of 370 peptides corresponding to 73 known or predicted transcripts. The peptides are marked as rectangles and are designated as JHU_xxxx where JHU stands for Johns Hopkins University and xxxx is the serial number assigned to the peptide. Clicking on the peptide accession numbers takes the user to a page hosted by our laboratory where additional details such as the peptide sequence are provided .

### Use of peptide sequences to validate known transcripts at the protein level

Transcripts annotated as known transcripts are generally those for whom a full length cDNA exists. However, it is possible for transcripts to exist without being translated, as pseudogenes are also known to be transcribed [9,10]. Here, we describe the use of peptide sequences obtained by tandem mass spectrometry to assist in the annotation and confirmation of exons of known transcripts. Figure 2 demonstrates how we were able to obtain peptide evidence for two of three exons of a known transcript encoding D7-related protein 1 whose cDNA was recently isolated [11,12]. Additional file 1 lists 172 peptides that led to validation of exons from 23 known transcripts.

### Use of peptide sequences to validate exons in novel transcripts

**Figure 3:**
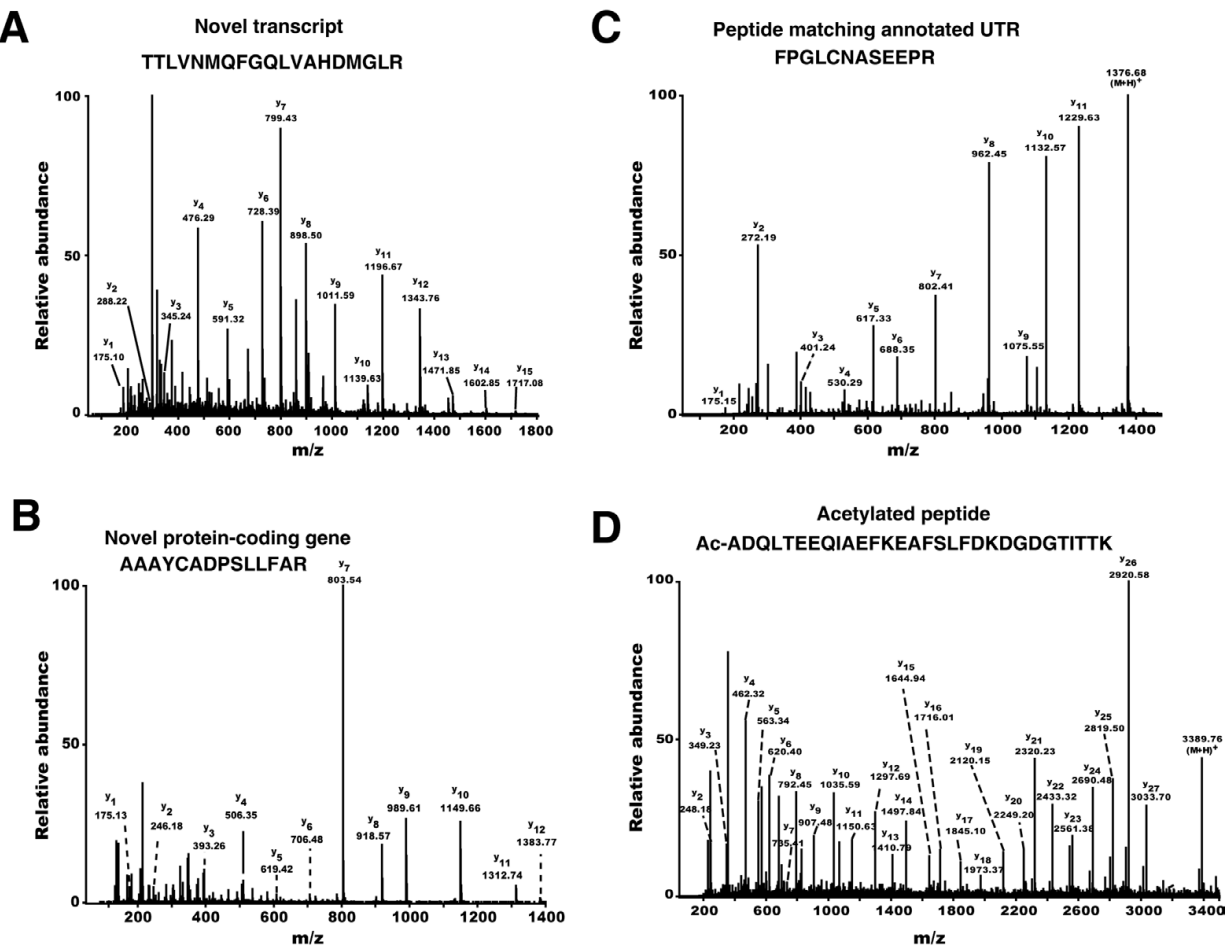
**MS/MS spectra in four different instances that were used for annotation of *An. gambiae *genome**. (A) MS/MS spectrum of a peptide, whose sequence validated an exon of a novel transcript encoding a peroxidase family of proteins ([ENSANG:P00000000593]); (B) MS/MS spectrum of a peptide used to identify a novel protein-coding gene. (C) MS/MS spectrum of a peptide that maps to a predicted UTR of a known transcript encoding Antigen 5-related 1 protein. (D) MS/MS spectrum corresponding to a peptide that is acetylated at its N-terminus. The acetyl moiety is denoted by Ac.

**Figure 4:**
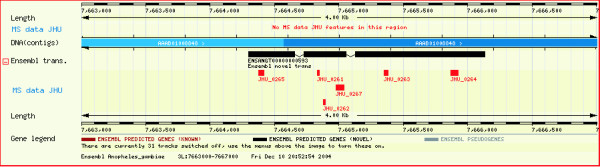
**A screenshot depicting validation of a novel transcript using 4 mass spectrometry-derived peptide sequences. **The figure shows a novel transcript ([ENSANG:T00000000593]) on chromosome 3L with the red rectangles on 'MS Data' track corresponding to peptide sequences obtained through mass spectrometry.

Novel transcripts generally refer to those obtained by gene prediction programs and might or might not have corresponding cDNA evidence. Often, a large number of transcripts are predicted from genome annotation pipelines and it would be helpful if one could easily determine if a transcript was not only expressed but also if the translated protein was present. For instance, in the case of the *An. gambiae *genome, there is a large degree of non-overlap in predicted transcripts between two different sets of annotations. We used the peptides that we identified and searched against the transcripts predicted by Ensembl. Figure 3A illustrates an MS/MS spectrum that led to identification of a peptide as TTLVNMQFGQLVAHDMGLR (Table 2- protein [ENSANG:P00000000593]), which matched the third annotated exon of a novel transcript predicted to encode a novel member of the peroxidase family of proteins. This peptide also matched another transcript as well ([ENSANG:P00000028058]). In cases where a peptide matched more than one genomic sequence, we have annotated all such regions, as we are unable to unambiguously determine the genomic sequence that codes for the peptide. Figure 4 shows validation of all 3 exons of this predicted transcript. Additional file 2 lists a total of 198 peptides that were used to validate the presence of proteins encoded by 50 novel transcripts.

**Table 1:** Proteins with the identified cSNP.

	**Protein Accession #**	**Protein name**	**Peptide sequence**	**Amino acid change**
	[ENSANG:P00000029569]	Similar to SGS4	SFA**S**DGTDVTVR	**A→S**
	[ENSANG:P00000025580]	D7-related 3 protein precursor	CNAEAEKV**H**TSSK	**D→H**
	[ENSANG:P00000012716]	Putative 5'-nucleotidase precursor	VPYDTKYDT**V**EGDYPLVVK	**I→V**
	[ENSANG:P00000000593]	Peroxidase precursor	LLPAEYGDGV**S**VPR	**Y→S**
	[ENSANG:P00000017522]	TRIO protein	S**Q**NPASP**A**GSLGGKDVVSK	**L→Q**
				**T→A**

**Table 2:** List of peptide sequences shown in Figures 3 and 6B.

**Protein Accession #**	**Protein name**	**Peptide sequence**	**Figure **#
[ENSANG:P00000000593]	Peroxidase family of proteins	TTLVNMQFGQLVAHDMGLR	3A
-	Novel protein-coding gene	AAAYCADPSLLFAR	3B
[ENSANG:P00000021028]/ [ENSANG:P00000023200]	Antigen 5-related 1 protein	FPGLCNASEEPR	3C
[ENSANG:P00000012700]	Similar to calmodulin1	ADQLTEEQIAEFKEAFSLFDKDGDGTITTK	3D
[ENSANG:P00000018280]	D7 protein family	FVDLSR TLNEANSR VIDCIFR IYAAMPQIK KVIDCIFR IPVQHEAYK KIWGGYNKK LYHGTVEGAAK GESFFAYCAK NYELSGSSQFK KLYHGTVEGAAK NYELSGSSQFKK CYEDHLPAGSSR ALDPEQALYVYK QKGESFFAYCAK SERIPVQHEAYK GRNYELSGSSQFK LEPNDAVTHCYAK VYEGPEQVKEEMK ALDPEQALYVYKR GRNYELSGSSQFKK	6B

### Identification of a novel protein-coding gene through peptide sequence data

**Figure 5:**
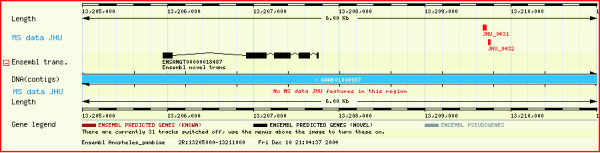
**A screenshot depicting identification of a novel gene. **Two peptide sequences AAAYCADPSLLFAR and MVVDGTFLR were mapped onto the forward strand of chromosome 2R, whose scaffold coordinates are 3012881–3012922 (JHU_431) and 3012836–3012862 (JHU_432), respectively. There are no known or novel transcripts where these two peptides matched as shown

In our analysis, we found several high quality MS/MS spectra that did not match any protein or predicted transcripts. These could arise from novel gene that were not predicted, or be due to a modified peptide. Figure 3B shows one such MS/MS spectrum that corresponds to the peptide sequence AAAYCADPSLLFAR (Table 2- Novel protein-coding gene). When the data was searched against the *An. gambiae *genome database, we found in addition to that peptide, another one that aligned perfectly to a region in the genome which had no known or predicted genes (Figure 5). Both of these peptide matches are unique in the *An. gambiae *genome. From these data, we conclude that we have identified a novel protein-coding gene although more detailed studies will be required to ascertain the exact structure of this gene.

### Correction of transcripts using peptide sequence data

**Figure 6:**
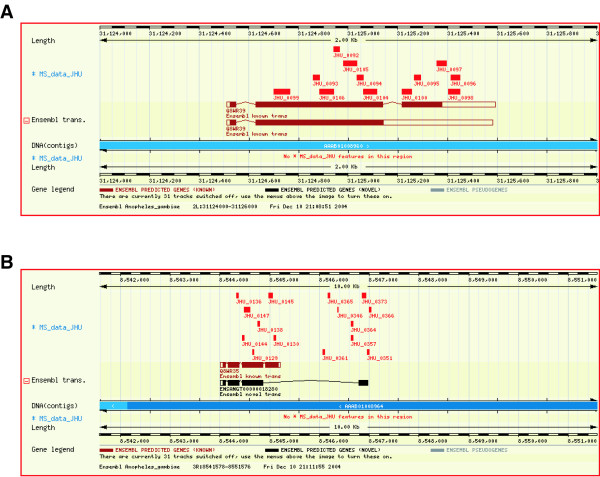
**A screenshot depicting the correction of annotation using peptide sequence data. **Panel A – Ensembl known gene [ENSANG:G00000018539] has two transcripts. Three peptides (JHU_0096, JHU_0097 and JHU_0098) align to the untranslated regions of both transcripts in this region. Panel B – Peptides are mapped onto the intronic regions of the Ensembl novel transcript [ENSANG:T00000018280]

Because of the limitations of gene prediction programs, exons can be missed entirely or their boundaries might be annotated incorrectly. This implies that errors in exon identification can also lead to erroneous assignments of coding exons as non-coding exons (untranslated regions) [13,14]. Figure 3C shows an MS/MS spectrum of a peptide corresponding to the sequence, FPGLCNASEEPR (Table 2- protein [ENSANG:P00000021028]/ [ENSANG:P00000023200]), which matched an annotated 3' UTR of Antigen 5-related 1 protein whose cDNA was described recently [12]. Figure 6A shows that the peptide FPGLCNASEEPR (JHU_0096) and two other peptides (JHU_0097 and JHU_0098) are located just downstream of the stop codon in one of the known transcripts. A closer examination of the Ensembl transcript in which this region was annotated as a UTR ([ENSANG:T00000021028]) revealed that it was erroneously marked as a UTR as there was no stop codon where the annotated coding region apparently ended. This explains why we were able to find the above-mentioned peptides. Further, we found that there was another transcript deposited in GenBank in which this region was annotated correctly as coding region (GenBank accession # Y17702). This other GenBank transcript was not seen in the genome browser view as it was not present in the Ensembl database. We hope that studies such as ours will help identify such errors and facilitate their correction in various databases. Figure 6B shows several peptides (Table 2- protein [ENSANG:P00000018280]) that matched an annotated intron in a novel transcript encoding a novel member of the D7 protein family. This is likely due to a false negative prediction for an exon in this genomic region.

### Use of mass spectrometry data for annotation of translational start sites

Proteins generally undergo proteolytic cleavage of their N-termini by aminopeptidases, *in vivo*, which results in removal of one or more amino acids from the N-termini [15]. In most cases, this is followed by addition of an acetyl moiety to the amino terminus of the processed protein often referred to as a 'blocked' N-terminus, which cannot be sequenced easily by the traditional Edman method. In this study, we found two acetylated peptides that matched two different predicted transcripts. The peptide sequence Ac-ADQLTEEQIAEFKEAFSLFDKDGDGTITTK, whose MS/MS spectrum is shown in Figure 3D (Ac refers to the acetyl moiety), corresponds to a novel transcript (Table 2- protein [ENSANG:P00000012700]). The predicted protein is orthologous to rabbit calmodulin (93% identity), which has been observed to contain a blocked amino terminus [16]. We found another N-terminally acetylated peptide sequence, Ac-STVDKEELVQK, which corresponds to another novel transcript ([ENSANG:P00000009311]) predicted to encode a protein very similar (96% identity) to a chaperone found in *D. melanogaster*. In both of the above-mentioned cases, the amino terminal methionine residue was cleaved and the newly exposed amino terminus was acetylated. Thus, we were able to validate the assignment of the translation initiation codons for these two predicted transcripts which is not always straightforward as it has been shown that, contrary to popular beliefs, the most 5' AUG codon is not used for translation initiation in a large proportion of cases [17,18]. We should also note that our strategy was not designed to enrich for N-termini of proteins. If such strategies were to be used in conjunction with mass spectrometry, a large number of N-termini could be assigned in a single experiment.

### Identification of sequence polymorphisms

**Figure 7:**
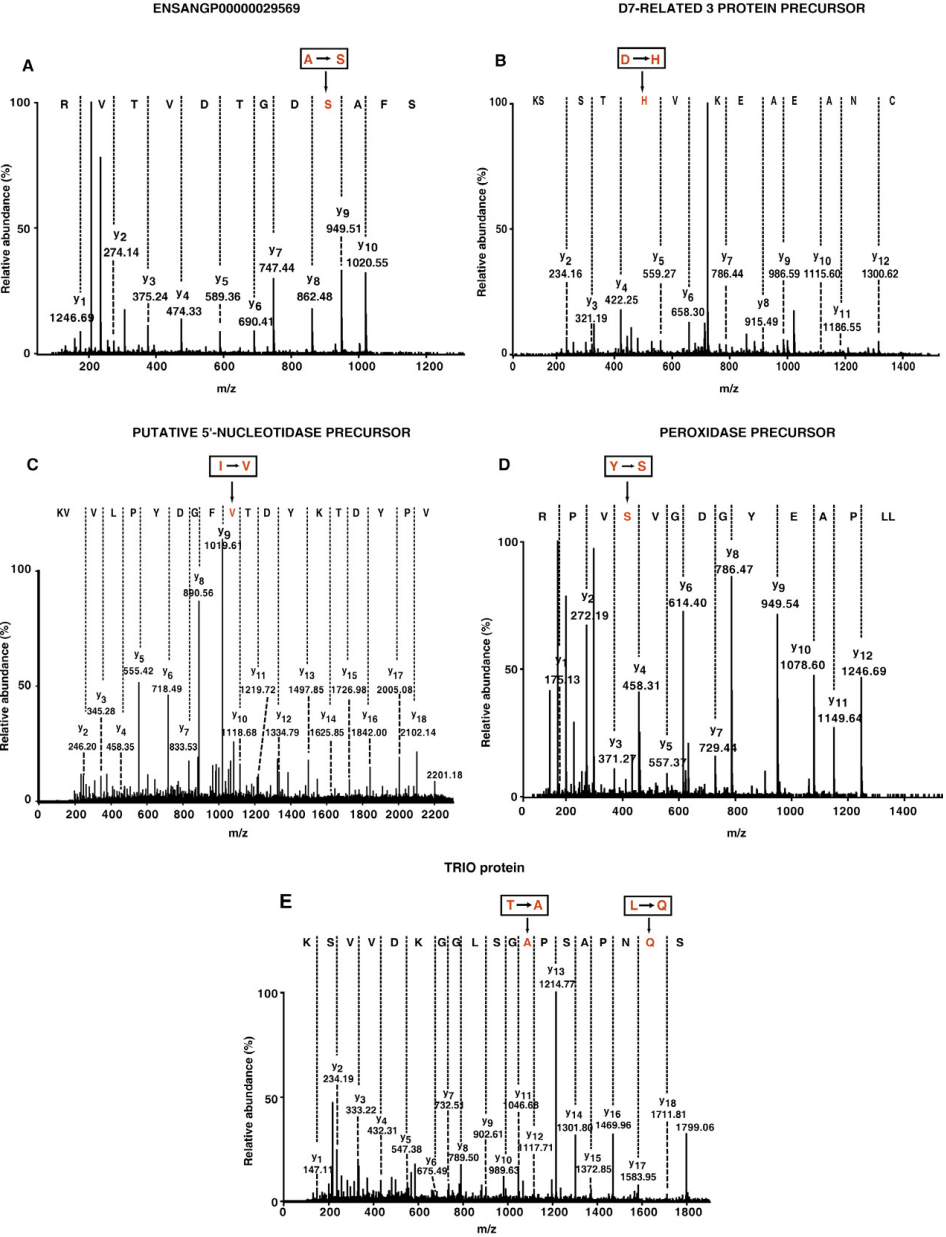
**MS/MS spectra of five different peptides that identify coding SNPs. **(A) The amino acid change Ala→ Ser is shown in the peptide SFASDGTDVTVR that matches the protein [ENSANG:P00000029569]. (B) The sequence of the peptide CNAEAEKVHTSSK that matches the D7-related 3 protein precursor ([ENSANG:P00000025580]) shows the amino acid change Asp→His. (C) The peptide VPYDTKYDTVEGDYPLVVK corresponding to the protein putative 5'-nucleotidase precursor ([ENSANG:P00000012716]) presents the amino acid change Ile→Val. (D) The amino acid change Y→S is identified in the peptide LLPAEYGDGVSVPR that corresponds to the protein peroxidase precursor ([ENSANG:P00000000593]). (E) Two changes L→ Q and T→A occur in the same peptide SQNPASPAGSLGGKDVVSK that corresponds to the TRIO protein ([ENSANG:P00000017522]). The amino acid changes representing the cSNPs for the five proteins are shown in rectangle.

In *An. gambiae *genome, 444,963 single nucleotide polymorphisms have been reported [1]. In this study, we identified six sequence polymorphisms. We carried out a search of the mass spectrometry-derived data against Ensembl *Anopheles gambiae *protein database using the X! Tandem algorithm. We found four cSNPs in four different proteins ([ENSANG:P00000029569], [ENSANG:P000000000593], [ENSANG:P00000012716], [ENSANG:P00000025580]) as shown in Table 1. All identified SNPs are novel and are consistent with being single nucleotide substitutions. Manual inspection of the mass spectra corresponding to the SNPs was carried out and through the clear presence of y ion series (as shown in Figure 7), we attributed the mass difference to amino acid change and eliminated the possibility of post-translational and other modifications. From our manual data analysis, two more SNPs were identified on a peptide derived from the Trio protein (Table 1). The sequence of one of the tryptic peptides that occurs in this protein obtained from translation of the genomic sequence is S**L**NPASP**T**GSLGGK. However, we found another peptide, S**Q**NPASP**A**GSLGGKDVVSK (Figure 7E), which contained two sequence polymorphisms relative to the genomic sequence (the amino acids that are different are in bold). This longer peptide is similar to the former one but contains a tryptic miscleavage and matches Trio protein variant found in NCBI nr database (GenBank Accession # AAL68795) but not in Ensembl. Both of these amino acid substitutions (leucine to glutamine and threonine to alanine), could be explained on the basis of a single base pair changes and hence are likely to be due to cSNPs. Interestingly, Ensembl annotations catalog eight SNPs that occur in Trio protein including three non-synonymous and five synonymous SNPs. These do not include any SNPs that could explain the polymorphisms that we have observed. Thus, it seems that we have identified polymorphisms that arise from cSNPs not yet been obtained by other methods. Thus, mass spectrometry-derived data can be used to complement genomic methods for cSNP identification as well.

## Conclusion

One of the basic components of the annotation of any genome is an accurate representation of protein-coding genes. However, even this seemingly simple task is quite difficult. Here, we demonstrate that mass spectrometry is a powerful tool for annotating protein-coding regions in genomes. Here we report a pilot study to annotate the *An. gambiae *genome. Using mass spectrometry-derived data, we validated the physical existence of 23 known and 50 predicted transcripts at the protein level and confirmed the N-termini of proteins encoded by two predicted transcripts based on N-terminal acetylation. We also identified two sequence polymorphisms based on peptide evidence that were not annotated as SNPs in the databases. Thus, mass spectrometry is a valuable complementary method for initial discovery of locations of non-synonymous SNPs. Importantly; we also identified a novel gene that was not predicted by automatic annotation pipelines at all. The task of assigning translational start sites is fraught with errors especially in the absence of transcript data. Similarly, UTRs can also be wrongly assigned. Using MS/MS data, we corrected the translational start sites and UTR assignment of proteins, which would otherwise be difficult, or impossible using molecular biology based methods. Our MS/MS derived peptide sequence data has been uploaded onto Ensembl DAS server and can be visualized using the Ensembl genome browser. In summary, we have demonstrated how mass spectrometry-derived data can be used to refine the annotations of a complex eukaryotic genome and share them with the biomedical community.

## Methods

### Mass spectrometric analysis

Salivary glands from female *An. gambiae *(G-3 strain) were homogenized and subjected to digestion with trypsin as described previously [19]. The tryptic peptides were subjected to LC-MS/MS and analyzed on a quadrupole time of flight mass spectrometer (QTOF US-API, Micromass, UK) as described [19]. A total of 16 LC-MS/MS runs were carried out and the 3,967 MS/MS spectra acquired were searched against both protein and genome databases, this led to identification of 369 unique peptide sequences. The acquisition and deconvolution of data were performed on a MassLynx Windows NT PC data system (version 4.0).

### Data analysis

The *An. gambiae *genome and proteome database (release 16.2) was downloaded from the Ensembl ftp site . Mass spectrometric data searches were performed using Mascot version 1.9 installed on a Linux cluster [20] against the NCBI non-redundant (nr) database as described earlier [21] The following settings were used: a) trypsin as the specific enzyme (al1ow up to 2 missed cleavages); b) peptide window tolerance (error window on experimental peptide mass values) ± 0.4 Da; and c) fragment mass tolerance of ± 0.3 Da. Moreover, during the searches, N-terminal acetylation, oxidation of methionine and carbamidomethylcysteine modification were the three amino acid modifications allowed. Searches were also carried out against the genome database. For this purpose, the large genome sequence files in FASTA format were trimmed into 100 kb long sequences since Mascot cannot deal with large genomic sequences. Same set of parameters were used for genome search as used for NCBInr search. Only peptides with a Mascot score greater than 30 and containing a sequence tag of at least four consecutive amino acids were considered in this study. The spectra were further investigated and verified by manual interpretation. Any peptide hits that matched transcripts labeled known or novel were investigated further using Ensembl browser. This included validation of existing exons, correction of intron-exon boundaries and mapping of N-termini of mature proteins. All peptide matches to the genome were compared with matches to the non-redundant protein database. Those peptides that did not match any entry in the nr database were analyzed further. This allowed identification of novel genes that are not predicted by gene prediction algorithms or correction of regions annotated as introns or untranslated regions.

In our study, if a peptide maps to more than one transcript, we have assigned such peptides to all of the corresponding transcripts. However, for the purpose of counting the number of transcripts/proteins that we have identified, we have included only the assignments of those peptides that had matches to only one transcript.

In order to identify the presence of potential cSNPs, we utilized the "point mutations" feature in X! Tandem, which allows the user to identify single amino acid changes in peptides. We searched the data against Ensembl Anopheles protein database using the X! Tandem 2 release search algorithm installed on a Linux cluster [22]. The searching parameters were the same as described above for search using Mascot.

### Use of Distributed Annotation System (Das)

The Distributed Annotation System provided by Ensembl was used to visualize the peptides in the context of genome and to share our annotations with the community. A genome database search was carried out using the peptides to determine the corresponding regions in the genome for each peptide. The positions of these peptides were obtained by implementing scripts written in Python programming language. These scripts parse the output files obtained by searching genome using TBLASTN algorithm. The genomic coordinates were packaged into a tab delimited format necessary for uploading onto the DAS server at Ensembl. Whenever a user chooses a 'MS data JHU' as a DAS server source, the peptide and its coordinates on the genome are mapped onto the browser and visualized on a separate track.

## List of abbreviations

*An. gambiae*: *Anopheles gambiae*

NCBI: National Center for Biotechnology Information

EBI: European Bioinformatics Institute

Mbp: Mega base pair

DAS: Distributed Annotation System

UTRs: Annotated untranslated regions

cSNPs: Coding single nucleotide polymorphisms

LC-MS/MS: Liquid chromatography nanoelectrospray tandem mass spectrometry

MS/MS: tandem mass spectrometry

JHU: Johns Hopkins University

## Authors' contributions

DK carried out the mass spectrometric analysis, database search and interpretation of the mass spectrometry-data and drafted the manuscript. SP and RR carried out the genome data analysis for inclusion in the Distributed Annotation System (DAS). RR carried out additional genome data analysis to identify potential cSNPs. JZ assisted in the mass spectrometry data analysis. BO dissected the salivary glands from female *Anopheles gambiae*. NK supervised the mosquito experiments and helped with design of the study and the manuscript. AP conceived the study, coordinated and assisted in drafting the manuscript. All authors read, endorsed and approved the final version of the manuscript.

## Supplementary Material

Additional File 1 A list of peptides used to validate annotated exons in known transcripts. A list of peptide sequences obtained by tandem mass spectrometry that were used to validate the presence of protein-coding exons in known transcripts in the Ensembl database. Click here for file

Additional File 2 A list of peptides used to validate novel transcripts. A list of peptide sequences obtained by tandem mass spectrometry that were used to validate protein-coding exons in novel transcripts in the Ensembl database. Click here for file
