# Supplementary material for: Genome annotation of Anopheles gambiae using mass spectrometry-derived data
Source: BMC Genomics. 2005 Sep 19;6:128. doi: 10.1186/1471-2164-6-128 (PMC1249570; doi:10.1186/1471-2164-6-128)
Supplement: Additional File 1 — A list of peptides used to validate annotated exons in known transcripts. A list of peptide sequences obtained by tandem mass spectrometry that were used to validate the presence of protein-coding exons in known transcripts in the Ensembl database. [file 1471-2164-6-128-S1.doc]

**Additional File 1**

**A list of peptides used to validate annotated exons in known transcripts**.

|  | **Ensembl Accession #** | **Peptide** |
| --- | --- | --- |
|  | [ENSANG:P00000017522] | 1. ALEPLQK 2. QVLDAWK 3. FNTPIDGK 4. GWLSVSQQEK 5. NSFATLVSIAR 6. HFPDHFDTLR 7. TLFEPMKADLAK 8. SMYDLIGQLVQSSK 9. LSQLAHAMPSLVDVK 10. AHPDLQQSVDDLMAK 11. SPTGSLGGKDVVSK 12. SLNPASPTGSLGGK |
|  | [ENSANG:P00000015382] | 1. VLSIDVR 2. LNVAQVAGLR 3. STDPFPALPR 4. VFHTVQELR 5. VVNESNLVGGR 6. AAEEGDTCIAGIAR 7. NLNDGVSYEPLNR 8. IAQTTVDLVQATCR |
|  | [ENSANG:P00000016300] | - - 1. ELEPWR     2. ECIAGIAR     3. SFGKYVGR     4. TMFQEKR     5. VGVIGALYDK     6. IPTVVANLEK     7. GLAPYLAELEK     8. VQSIDIVGANGAK     9. MKIPTVVANLEK     10. YDTIEGDYPLVVK     11. SSTCTNSKECIAGIAR     12. VTGPLDSDVLIEYVR     13. LSGADLWSAIDHSFTLDDEFR     14. DQIYYVVVPSYLADGKDGFAMLK     15. TKNPLYLNAGDNFQGTLWYNLLR     16. LSGADLWSAIDHSFTLDDEFRYNTAQVSGMAVVADLSK |
|  | [ENSANG:P00000018328] | 1. VFDLMELK 2. KVFDLMELK 3. YTAEFVQIMK 4. LYDPLNIIELDK 5. CIGECVQVPTSER 6. LYDPLNIIELDKR 7. ALDFVYEDGRGDYHK 8. YEIIEGPEMDKHIHCVMR 9. RYEIIEGPEMDKHIHCVMR |
|  | [ENSANG:P00000018371] | 1. NIGDSLKDR 2. NAVDYNELLK 3. ESVLLELLQR 4. VDSGVNHAANMK 5. MQTSDPFDMNR 6. VDSGVNHAANMKK 7. QYTPVSSDDMDK 8. VAALIKEIDDGLC 9. ANTFYTCFLGTSSLAGFK 10. ESTVEECEKNIGDSLKDR 11. KANTFYTCFLGTSSLAGFK 12. ANTFYTCFLGTSSLAGFKNAVDYNELLK 13. CVTEASTSGSDKKANTFYTCFLGTSSLAGFK 14. QYTPVSSDDMDKHMQCVLEVVGFVDGNGEVK 15. QYTPVSSDDMDKHMQCVLEVVGFVDGNGEVKESVLLELLQR |
|  | [ENSANG:P00000018330] | 1. YAVDYVELLR 2. MVDPDGDHAGSMK 3. MVDPDGDHAGSMKK 4. ANTFYTCFLGTSSAQAFK 5. AGKLDMGTTFNAGQVSALMK |
|  | [ENSANG:P00000018340] | 1. SQLCEIR 2. SQLCEIRK 3. VFDTVELVK 4. CGGQTQHLPVGK 5. CGGQTQHLPVGKR 6. CLVESTSGEAFKK 7. KLPALSQYSSVVDK 8. ALDFVRPDGTGDYHK 9. LLDTPDMDKHMDCVMK |
|  | [ENSANG:P00000021028] | 1. SCNYGHDR 2. IRPYPSAVK 3. FTEKDLIHK 4. VGCSMWYWK^1^ 5. FPGLCNASEEPR^1^ 6. SVYQSGPTASQCK^1^ 7. NSKFPGLCNASEEPR^1^ 8. GGPHVGCNPPSSSGGPTCQGK 9. YPSSYSGKPIGHFTQIASDR 10. KYPSSYSGKPIGHFTQIASDR 11. FVSSWWSEYLDARPEHVR 12. KFPYAGQNIAITQFFGYR 13. MPTLTWDPELASLADANAR |
|  | [ENSANG:P00000001387] | 1. FQSSAVMALQEASEAYLVGLFEDTNLCAIHAK |
|  | [ENSANG:P00000018321] | 1. GVEADRYVQCFMTALGFADESGSIQR |
|  | [ENSANG:P00000019455] | 1. FCTLCDTR 2. SMHDVLCDR 3. HFCECKETR 4. VYCGHLDCTR 5. SMHDVLCDRIDQAFLEQ 6. EPLPYMYACPGTEPCQSSDR 7. ETREPLPYMYACPGTEPCQSSDR 8. HFCECKETREPLPYMYACPGTEPCQSSDR |
|  | [ENSANG:P00000012895] | 1. HEQNIDCGGGYLK 2. TWVQSEHKGVEYGK 3. FYNDAEADKGLQTSQDAR |
|  | [ENSANG:P00000025174] | 1. SDLEPEVR 2. NAMDCVFR 3. IYEAKPEIK 4. SANYGYLAMGK 5. NAFYFHELR 6. DYELADSAEFR 7. KIYHGTVDSVAK 8. LRDYELADSAEFR 9. QEESFFAYCAK 10. CTLAGLQMYDEK 11. KQEESFFAYCAK 12. IRDYELADSAEFR 13. ALDPEEAWYVYER 14. YMDDSGLKVDEVVR 15. DFNLINKSDLEPEVR 16. TNTFKPETVPVQHEAYK 17. SVLASCTGTQAYDYYSCLLNSPVKEDFR 18. VNELQQALSSLNAGSGSCAEVFNAYLPVHNK 19. SFTEVESSKVNELQQALSSLNAGSGSCAEVFNAYLPVHNK |
|  | [ENSANG:P00000007337] | 1. **MMLLQINQR** 2. **LAFLSQNADAR** 3. **ELDKCESFVK** 4. **SLVLMSVQGGASK** 5. **DSYLAMIAALDLGK** 6. **DANRDYDHYLQESK** |
|  | [ENSANG:P00000018379] | 1. QEATANDPQHDCSDSIQR |
|  | [ENSANG:P00000004315] | 1. DKPDIDPVDFLVDVIK |
|  | [ENSANG:P00000019451] | 1. MAMTGGGILGGIFSAL |
|  | [ENSANG:P00000017327] | 1. QHIANLLEFIR 2. DRQHIANLLEFIR 3. QFGGTLLPLLNSAQVK 4. KDPATAGLVTEVQNIFGR 5. YDLFLLDEHHPQLFDLLFDR 6. ALLTGANRYDLFLLDEHHPQLFDLLFDR |
|  | [ENSANG:P00000019055] | 1. GYSFTTTAER 2. EITALAPSTMK 3. MQKEITALAPSTMK 4. VAPEEHPVLLTEAPLNPK |
|  | [ENSANG:P00000019154] | 1. LFEQIGAK^1^ 2. EIVQFATK^1^ 3. FFADHLAK 4. LKGQFADFDR 5. ELEWKDELVK 6. TLYQLLQPEIMK^1^ |
|  | [ENSANG:P00000022875] | 1. YWCDSGYGSNDCK |
|  | [ENSANG:P00000023463] | - - - 1. LNHWHDDHPFLLETVAK       2. GQWKPDQIVSAIQDGYHVNPTIMEHLLEFVR       3. AHLEEILPTLALETEK       4. TVEQTGKSSAELVR       5. LFEIASLFK       6. FAAIEQLLK       7. LVPELQRK       8. DNFLFR |
|  | [ENSANG:P00000019156] | 1. QLQTSLATIELK 2. QFDICEQFIGR 3. GKFATFAK 4. DIPSVEER 5. QLEELRGK 6. SLYQLAQR 7. VLLSQIGDR 8. QLDALTDSR 9. QLYDDLVR 10. VTIYQDQR 11. RSLYQLAQR 12. EDLPVYQANR 13. LKEQVANGNFK 14. VLLSQIGDREK 15. GKNYQHYLSESR 16. FVPYANALPQPAQR 17. GKNYQHYLSESRK 18. AAAGPAPDPSSQFCQQLLDDAQR 19. SLHGGSMQPDGTCDNLWESFLSQFHQVR |

^1^Peptide matches to annotated UTRs
