# Supplementary material for: Genome annotation of Anopheles gambiae using mass spectrometry-derived data
Source: BMC Genomics. 2005 Sep 19;6:128. doi: 10.1186/1471-2164-6-128 (PMC1249570; doi:10.1186/1471-2164-6-128)
Supplement: Additional File 2 — A list of peptides used to validate novel transcripts. A list of peptide sequences obtained by tandem mass spectrometry that were used to validate protein-coding exons in novel transcripts in the Ensembl database. [file 1471-2164-6-128-S2.doc]

**Additional File 2**

**A list of peptides used to validate novel transcripts.**

|  | Ensembl Accession # | Peptide |
| --- | --- | --- |
|  | [ENSANG:P00000029569] | 1. NLLGGTDGPK 2. ELNELLVAAK 3. ASNTISLVTVR 4. HAVVYDTQTNNPQEIQVVAPR |
|  | [ENSANG:P00000027299] | 1. LLDEDTIR 2. SQNGNSFLTLSQPEHK 3. SFLTTDCTNPSLCHGR 4. TLYGDMNDDLPFVCGKR |
|  | [ENSANG:P00000015826] | 1. LGIMEDPSNR 2. WLNDVALKDK 3. QDLVNNLGTIAK 4. QDNIYFIAGPNR 5. GVVDSDDLPLNVSR 6. NKEYTSLSDYVAR 7. EGFTLNESEESKAR 8. KVSDVTDEEYTEFYK |
|  | [ENSANG:P00000027211]/ [ENSANG:P00000018385] | 1. FSVSGYPTLK 2. LKDEAVSIVK 3. LTPTLEELGTK 4. SEPVPESNDGPVK 5. DLLTVEAFEAFLK 6. FSVSGYPTLK |
|  | [ENSANG:P00000026077]/ [ENSANG:P00000011666] | 1. ELETVEAAEEFLKENNVAVVGFFK 2. TLEGFVK 3. ALAPEYAK 4. KDEVPSLR 5. FDEVAFDK 6. QLVPIYDK 7. TEDGVLVLTK 8. ILEFFGMK 9. ILEFFGMKK 10. VDATVEPELAEK 11. IDATANELEHTK 12. QLVPIYDKLGEK |
|  | [ENSANG:P00000019887] | 1. DAGTISGLNVLR 2. TTPSYVAFTDTER |
|  | [ENSANG:P00000020184] | 1. DDLFNTNASIVR |
|  | [ENSANG:P00000024604] | 1. APGIIPR 2. AVDSLVPIGR 3. ALLQQIASEGK |
|  | [ENSANG:P00000011028] | 1. AYYGVLKK |
|  | [ENSANG:P00000018716] | 1. FAEIVQLR |
|  | [ENSANG:P00000017331] | 1. AKLEEEIR |
|  | [ENSANG:P00000021319] | 1. GYADFCVR 2. GIVDESVTGVHR |
|  | [ENSANG:P00000021797] | 1. NIVWNIDNPK |
|  | [ENSANG:P00000013302] | 1. LSNVFIIGK |
|  | [ENSANG:P00000011648] | 1. FINEQLYPVAK 2. AQLKDGAFPTKR |
|  | [ENSANG:P00000020140] | 1. DIAEAALAEAK |
|  | [ENSANG:P00000021424] | 1. GISQGLADNTVIAR |
|  | [ENSANG:P00000018478] | 1. AVENSGTVIGLR |
|  | [ENSANG:P00000022049] | 1. VLEQLTNQTPVYSK |
|  | [ENSANG:P00000011974] | 1. MNPDGLAALAR |
|  | [ENSANG:P00000021580] | 1. GDLGIEIPAEK |
|  | [ENSANG:P00000012700] | 1. ADQLTEEQIAEFKEAFSLFDKDGDGTITTK 2. SLGQNPTEAELQDMINEVDADGNGTIDFPEFLTMMAR |
|  | [ENSANG:P00000000593] | 1. ALDIQR 2. HFLFR 3. ARPLWNDEK 4. TTLVNMQFGQLVAHDMGLR 5. ILCDNTPGVAQMQQR 6. AFAGAININDHMFNPTVLER |
|  | [ENSANG:P00000002671] | 1. INVYYNEASGGK 2. AVLVDLEPGTMDSVR |
|  | [ENSANG:P00000003420] | 1. YGFVVDGTR |
|  | [ENSANG:P00000009311] | 1. STVDKEELVQK 2. NLLSVAYK 3. YLAEVATGETR 4. SVTETGVELSNEER |
|  | [ENSANG:P00000009989] | - 1. APGIIPR   2. QMSLLLR   3. AVDSLVPIGR   4. ALLQQIASEGK   5. VVDALGNAIDGKGEIK |
|  | [ENSANG:P00000010360] | 1. LSKPATYDQIK 2. GAAQNIIPAATGAAK |
|  | [ENSANG:P00000011259] | - - 1. VVTATDEADLAR |
|  | [ENSANG:P00000021793] | - - 1. NADDISQEEYGEFYK     2. GVVDSEDLPLNISR |
|  | [ENSANG:P00000011832] | 1. TSFFQALSIPTK |
|  | [ENSANG:P00000012072] | 1. NLLSVAYK 2. DAAENSLVAYK 3. YLAEFATGGDRK 4. VASMDVELTVEER |
|  | [ENSANG:P00000012893] | 1. VSTGQGDKVFAPEEISAMVLGK 2. LTPDDIER 3. MKETAEAYLGK 4. NELESYAYSLK 5. ELEDIVQPIIAK 6. ITPSYVAFTADGER 7. AKFEELNMDLFR 8. NQLTTNPENTVFDAK 9. IINEPTAAAIAYGLDKK 10. VTHAVVTVPAYFNDAQR |
|  | [ENSANG:P00000015289] | 1. KPSYLQR 2. YGVQNQLR 3. SMVQNFDYTK 4. TLADETAQCMR 5. VTDLEASFYASFSYNCHDHDQYSMECLEAAEPK |
|  | [ENSANG:P00000016630] | 1. TVISQSLSK 2. NMIGFYDMAR 3. LAEMPADSGYPAYLGAR |
|  | [ENSANG:P00000016676] | 1. LQCQDFLR |
|  | [ENSANG:P00000016868] | 1. VLDTGSPIR 2. IPVGAETLGR 3. VVDLLAPYAK 4. TIAMDGTEGLVR 5. IINVIGEPIDER 6. VALVYGQMNEPPGAR 7. IPSAVGYQPTLATDMGSMQER |
|  | [ENSANG:P00000017506] | 1. YTLYNVGSGPAVNVR |
|  | [ENSANG:P00000017682] | 1. FDWWER^1^ 2. TFMQLTAYR 3. GNFYQIYPR 4. TWLPVADGYR 5. GSAWQWSDKR 6. SFKDSDGDGIGDLK |
|  | [ENSANG:P00000018280] | 1. FVDLSR^3^ 2. TLNEANSR^3^ 3. VIDCIFR^3^ 4. SDLEPEVR 5. IYAAMPQIK^3^ 6. KVIDCIFR^3^ 7. SADYAFLLR 8. IPVQHEAYK^3^ 9. KIWGGYNKK^3^ 10. LYHGTVEGAAK^3^ 11. NAFDFHELR 12. GESFFAYCAK^3^ 13. NYELSGSSQFK^3^ 14. KLYHGTVEGAAK^3^ 15. NYELSGSSQFKK^3^ 16. CYEDHLPAGSSR^3^ 17. ALDPEQALYVYK^3^ 18. QKGESFFAYCAK^3^ 19. SERIPVQHEAYK^3^ 20. GRNYELSGSSQFK^3^ 21. LEPNDAVTHCYAK^3^ 22. VYEGPEQVKEEMK^3^ 23. ALDPEQALYVYKR^3^ 24. GRNYELSGSSQFKK^3^ 25. YMDDSGLKVDEVVR 26. GKVYEGPEQVKEEMK 27. DFNLINKSDLEPEVR 28. SVLASCTGTQAYDYYSCLLNSPVKEDFK 29. SVLASCTGTQAYDYYSCLLNSPVKEDFKNAFDFHELR |
|  | [ENSANG:P00000018372] | 1. VETGVLKPGTVVVFAPVNLTTEVK 2. QLIVGVNK 3. TIEKFEK 4. QTVAVGVIK 5. EVSSYIKK 6. IGGIGTVPVGR 7. TIEKFEKEAQEMGK |
|  | [ENSANG:P00000018531] | 1. IGSEVYHHLK 2. EALNLIQDAIAK |
|  | [ENSANG:P00000019238]/ [ENSANG:P00000027418] | 1. LFEQFK 2. LLPGADER 3. DYPEYFAR 4. QQWSAEGLDR 5. GFPDYEALYK 6. LPAYLSEVSAR 7. VEEPLYETLK 8. VEATVGQADAAVR 9. MVSFPNALPVGVQR 10. LAACVVSEHEQAYR 11. LAHETGRVEATVGQADAAVR 12. ALLETLLQHQGEQNNDVYLIR 13. YKPDHAQYEGK 14. WLAELQAGTFHEVVEFAR 15. VEEPLYETLKQQWSAEGLDR 16. VNVLQVSLQHDLPNLQEMVGEQR^2^ 17. HLIEQAWQYGAQLQHELMLTSMESDR 18. HLIEQAWQYGAQLQHELMLTSMESDRVQR |
|  | [ENSANG:P00000020171] | - 1. FVDIAIPCNTK   2. YAHYTQATPIAGR |
|  | [ENSANG:P00000020268] | - - - 1. SERIVEQR^3^ |
|  | [ENSANG:P00000021830] | 1. MGLTEFQAVK 2. LEATADKYNLQVR |
|  | [ENSANG:P00000028522]/[ENSANG:P00000022344] | 1. SFVVGR 2. ELDDGLIER 3. VDTKDKYLK^2^ 4. IKECFSSLDK 5. ECFSSLDKDVSAMVK 6. IKECFSSLDKDVSAMVK 7. EQELSDCIVDKR 8. LMNPTIDLVSTIEK 9. TNTSCGTEGTRELDDGLIER 10. ELDDGLIEREQELSDCIVDKR 11. ESEKSYEECSKDK 12. LMNPTIDLVSTIEKYSK 13. SYEECSKDKTNTSCGTEGTR 14. EGEEGAGSDDAVSGADDETEESKDDAEEDSEEGGEEGGDGASGGEGGEKESPR |
|  | [ENSANG:P00000022839] | 1. YNYEFDKIPR 2. MTGVASGSLDSSGNVVQNTAR |
|  | [ENSANG:P00000023320] | - 1. APDFVFFAPR |
|  | [ENSANG:P00000024159] | 1. GILAADESTATCGK 2. GILAADESTATCGKR |

^1^Peptide matches to annotated UTRs; ^2^Peptide “linking” two adjacent transcripts; ^3^Peptide matches to annotated introns
